# A New *Ferula* (Apiaceae) Species from Southwest Anatolia: *Ferula pisidica* Akalın & Miski

**DOI:** 10.3390/plants9060740

**Published:** 2020-06-12

**Authors:** Emine Akalın, Hüseyin Onur Tuncay, Bülent Olcay, Mahmut Miski

**Affiliations:** 1 Department of Pharmaceutical Botany, Faculty of Pharmacy, Istanbul University, Istanbul 34116, Turkey; onur.tuncay@istanbul.edu.tr (H.O.T.); bulentolcay@istanbul.edu.tr (B.O.); 2 Department of Pharmacognosy, Faculty of Pharmacy, Istanbul University, Istanbul 34116, Turkey

**Keywords:** *Ferula*, *Ferula pisidica*, new species, Turkey, Apiaceae, Anatolia, anatomy, chemotaxonomy

## Abstract

*Ferula pisidica* is a novel endemic species found in the vicinity of Karaman province of inner Mediterranean Region of Turkey. *F. pisidica* is morphologically distinct from *F. haussknechtii* and *F. brevipedicellata* by habit, sheaths, terminal leaf lobes, and mericarp size as well as by chemotaxonomic differences. The new species is described by morphological, carpological, ecological and phytochemical characteristics. Its relationships with the other related species and proposed conservation status will be reviewed.

## 1. Introduction

The Apiaceae is one of the largest family of Plant Kingdom and shows extreme diversity in Angiosperm groups [1,2]. It is possible to evaluate as another indicator fewer number of species in the genera (434 genera and 3780 species, average 8.7 species (APG III)) diversity and variety in the family.

*Ferula* L. species were widespread in the temperate regions of Euro-Asian continent surrounded by Canary Islands in the West, China and India in the East, North Africa in the South, and Central Europe in the North. With more than 185 species, the genus *Ferula* is the largest genus of Apiaceae family [2,3]

The genus *Ferula* species distributed in the Irano-Turanian region was classified by Boissier into three sections based on the number of their vittae and the shape of their petals: *Peucedanoides* Boiss. *Euferula* Boiss. and *Scrodosma* Bunge [4].

Korovin introduced a novel and wider taxonomical classification by inclusion of the leaf and floral characteristics of *Ferula* species. However, he underestimated the carpological characters of genus [3]. Korovin defined six subgenera and eight sections in his monograph. Furthermore, he has divided each section into series or hybrid (grex) groups. In contrast, Safina and Pimenov have emphasized the fruit anatomy of *Ferula* and *Peucedanoides* sections while pointing out the heterogeneity amongst the species [5].

Cumulative analyses of 90 *Ferula* species based on molecular characters yielded quite different results in comparison with Korovin’s taxonomical classification [6]. In addition to the morphological characters, life cycle features and chemical classification data were used in this study which led to the definition of 12 sections by Safina and Pimenov [5].

Boissier was the first botanist who dealt with the *Ferula* species growing in Turkey [4]. Later, Peşmen listed 18 *Ferula* species (one of them as an incompletely known species) without any taxonomical subdivision of the genus in his monograph of Turkish *Ferula* species [7]. A recent revision of *Ferula* species growing in Turkey was completed by Sağıroğlu and Duman, with the introduction of four new species (i.e., *F. coskunii* H. Duman & M. Sağıroğlu, *F. mervynii* Sağıroğlu & H. Duman, *F. brevipedicellata* Peșmen ex Sağıroğlu & H. Duman and *F. duranii* Sağıroğlu & H. Duman) the total number of *Ferula* species growing in Turkey has reached 22 [8,9,10,11].

Due to the wide morphological variations and potential inter-species hybridization, taxonomical classification of *Ferula* species’ is very difficult. In order to resolve taxonomical issues of the genus, several recent publications were focused on the phylogenetic analyses of *Ferula* species [12,13,14,15,16,17,18,19].

Recently, Panahi et al. compared nrDNA ITS and plastid DNA sequences of 153 samples from Irano-Turanian floristic region including some specimens from Turkey. Based on the data, a new classification system with four subgenera and 10 sections was proposed [17]. Results of this study were highly controversial and suggest the presence of intensive reticulate evolution for the *Ferula* specimens from Turkey. The Turkish *Ferula* species included in this study were classified under two subgenera: subgenus *Ferula* and subgenus *Narthex* (Falc.) Drude. *F. communis* L. and *F. tingitana* L. species were placed in the Section *Ferula* of Subgenus *Ferula*, *F. caspica* M. Bieb., *F. haussknechtii* H. Wolff ex Rech.f., *F. orientalis* L., *F. rigidula* Fisch. ex DC. were assigned to the Section *Peucedanoides* of Subgenus *Narthex*, *F. szowitsiana* DC. species was placed in the Section *Merwia* (Fedtsch.) Koso-Pol., and *F. coskunii* and *F. mervynii* were placed in the Section *Stenocarpa* of Subgenus *Narthex* along with *F. stenocarpa* Boiss. & Hausskn. ex Boiss., an endemic species from the Flora of Iran. These latter species were reconsidered as unusual species [17]. In another phylogenetic study that was performed simultaneously with the aforementioned paper, two monocarpic Turkish species; *F. drudeana* Korovin and *F. huber-morathii* Peşmen were included in Korovin’s Subgenus *Merwia,* whereas *F. anatolica* Boiss., *F. mervynii* and *F. coskunii* were identified as unassigned species [18]. These examples clearly illustrates the problems associated with the application of phylogenetic analyses to the taxonomical classification of Turkish *Ferula* species.

Except for *F. jaeschkeana* Vatke (i.e., chromosom number of 2n = 2x = 26), *Ferula* species are diploid with chromosome number of 2n = 22 and such information does not provide distinct taxonomical classification data [9,20,21].

Owing to the extremely large size of most *Ferula* species, majority of the herbarium specimens either contain fewer parts of the plant or selectively collected smaller plant samples that were not representative of the actual living specimen of species which inadvertently result in the incomplete description of many *Ferula* species. Furthermore, shape and anatomical characters of fruits and basal leaves were important criteria for the identification of species, and yet during the fruit formation phase basal leaves of some species disintegrates. Some type specimen of *Ferula* species only contain early or late development stage plant samples. In order to accomplish a correct description of a *Ferula* species, field observation of the living plants and collection of samples from several populations are very important.

**Figure 1:**
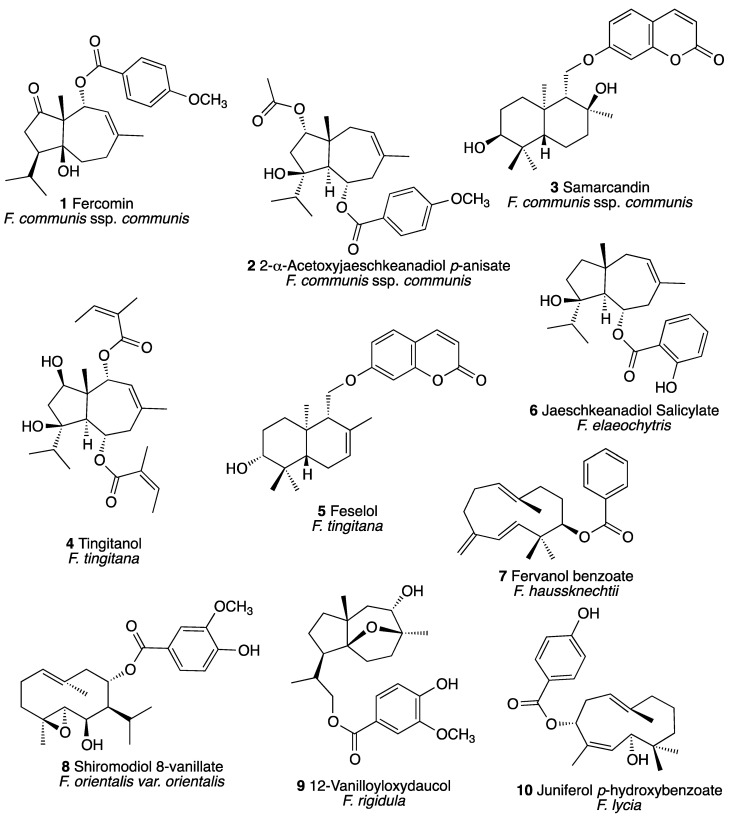
Various Sesquiterpenoid Compounds Isolated from the *Ferula* species Growing in Turkey.

Due to the difficulties encountered during the isolation and structure elucidation of complex sesquiterpenoid compounds from *Ferula* species, so far sesquiterpene based chemotaxonomic classification studies of *Ferula* species were not explored. Nevertheless, most significant secondary metabolites of *Ferula* species were sesquiterpenoid compounds that show unique chemical structure variations closely associated with their taxonomic status at the subgenera level. For example, *Ferula* species from Subgenus *Merwia* contain mainly sesquiterpene coumarin ethers and some sulfur containing compounds, on the other hand *Ferula* species from Subgenus *Ferula* yield both sesquiterpene coumarin ethers and sesquiterpene esters. In contrast, *Ferula* species from Subgenus *Peucedanoides* mainly afford sesquiterpene esters. Some of the major sesquiterpenoid metabolites isolated from the *Ferula* species growing in Turkey were shown in Figure 1. *Ferula communis* subsp. *communis* [22,23,24] and *F. tingitana* [25,26] from Subgenus *Ferula* yield several sesquiterpene coumarin ethers and sesquiterpene esters; *F. elaeochytris* Korov. [27], *F. orientalis* [28], *F. rigidula* [29], *F. haussknechtii* [30] and *F. lycia* Boiss. [31] from Subgenus *Peucedanoides* yield mainly daucane, apiene and germacrane sesquiterpene esters. Because of the presence of multiple chiral centers and variety of structural features such as mono-, di-, tricylic skeletons that vastly enhance their structural diversity, utilization of the sesquiterpenoid compounds as chemotaxonomic markers provide an unparalleled advantage over the use of classical phenolic chemotaxonomic markers such as flavonoids.

In consideration of the aforementioned criteria, the taxonomic status of *Ferula pisidica* was extensively evaluated based on the comprehensive field observations, taxonomic survey and in depth chemotaxonomical analysis. Although it has some closely allied species in the *Xeronarthex* Korov. Section of Subgenus *Peucedanoides* (see Section 3.2)*, F. pisidica* significantly differs from those species and has been confirmed as a new species.

## 2. Results

**Figure 2:**
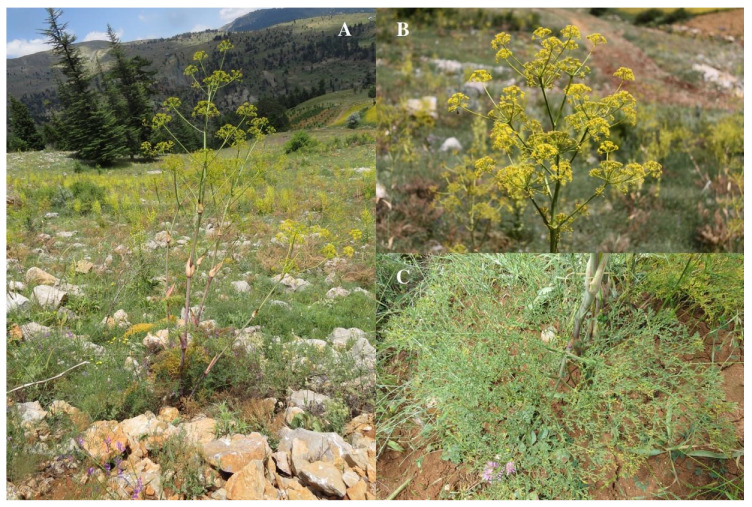
(**A**) Habit of *Ferula pisidica* in habitat. (**B**) Umbel. (**C**) Basal Leaf.

**Figure 3:**
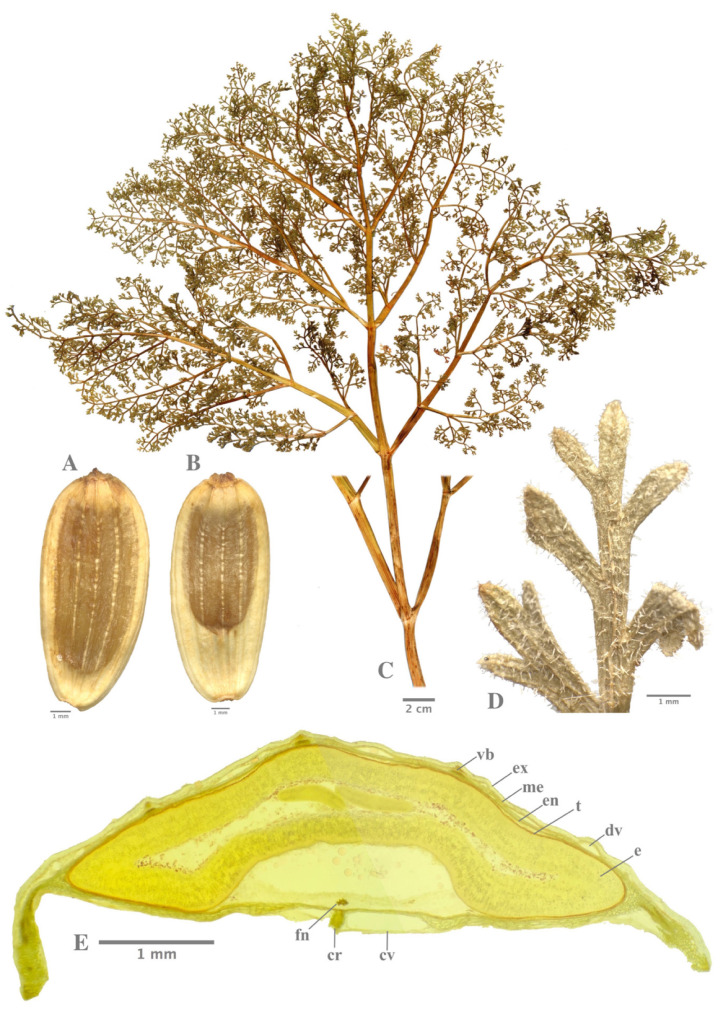
(**A**,**B**) General view of *F. pisidica* fruits. (**C**,**D**) Basal leaf of *F. pisidica*. (**E**) Cross section of mericarp of *F. pisidica* (cr carpophore, cv commissural vittae, dv dorsal vittae, e endosperma, en endocarp, ex exocarp, fn funicle, me mesocarp, t testa, vb vascular bundle).

*Ferula pisidica* Akalın & Miski *sp. nova* (Figure 2 and Figure 3)

Type: Turkey. C4 Antalya: Near Beyreli village, 1550 m, 26 June 2015, 36°50′24.8′′ N, 32°22′14.41′′ E, M. Miski, E. Akalın & S. Anıl. (holotype: ISTE 117051)

*F. haussknechtii* and *F. brevipedicellata* are the closely allied species. *F. haussknechtii* differs by its shorter and slender habit, narrower and smaller sheaths, *F. brevipedicellata* differs by terminal leaf lobes ((3–)7–12 mm), larger and wider sheaths.

The petiole of basal leaves is 20–32 cm long in the new species, *F. haussknechtii* basal leaves sessile and *F. brevipedicellata* basal leaves petiole is 10–20 cm long.

### 2.1. Description

Erect, green, perennial herbs, polycarpic, 1–3 stemmed, up to 250 cm tall, solid, 1–3 cm diameter at base, stem glabrous and sulcate. Root 3–5 cm width with thick woody tap root system. Fibrous collar which are old petioles remains on the base of the stem. Leaves green, mostly basal, scabrid-setulose. Basal leaves petiolate, 50–72 cm long, 30–85 cm wide. Petioles of basal leaves 20–32 cm long. Basal leaves sheaths 4–9 cm long, 1–3 cm wide, not swollen. Petiole, equal or shorter than length of lamina. Basal leaf lamina (blade) triangular-ovate in outline; 25–40 cm long, 30–85 cm wide; 5–6 pennate; terminal lobes 2–3, each lobe 1–4 mm × 0.5–1.5 mm, linear, apex obtuse.

Cauline leaves with petioles 20–35 cm long, 12–30 cm wide. Cauline leaves with big or large broadly ovate sheath, 5–10 cm long, 4–8 cm wide, swollen.

Inflorescence paniculate-corymbose, central umbels composed of fertile flowers, lateral umbels composed of sterile flowers. Central umbels sessile or peduncle 1.5 cm long, lateral rays ascending, 8–22 rays, lateral rays 6–10 cm long, central rays 2–4 cm long, bracts caducous in fruiting time.

Umbellules (7–)9–15 flowered; pedicel at fruiting 2–8 mm long; sepals caducous in fruiting time. Petals yellow, setulose-scabrid on dorsal surface, 1–2 × 1 mm long. Filaments 1.5–2 mm long, reflexed, anthers ±oblong, 0.5–0.75 mm long. Stylopodium conical in fertile flowers, depressed in sterile flowers. Styles up to 2.5 mm long in fruit.

Mericarps oblong, 8–14 mm × 4–9 mm, depressed dorsally; brown when ripe; dorsal ridges filiform, lateral wings 0.5–1 mm wide, inferior wings up to 5 mm or absent, styles up to 2.5 mm long, dorsal vittae 1–3 per vallecula, commissural (2–)4–6.

Flowering time is from May to June and in fruit from June to July.

### 2.2. Carpology

In transversal section, mericarps shapes are elliptical (Figure 3). Cuticula is thin and smooth. Exocarp consists of thick-walled isodiametric cells in a single line. Exocarp is cut in the commissural area of 2 mericarps. Vascular bundles are placed in the dorsal ribs and lateral wings as a group consisting of vascular bundles. Each vascular bundle upper side is accompanied by some sclerenchymatous tissue. Trachea and tracheids are not distinguished from each other in xylem. Dorsal vittae are 1–3 per vallecula (one of them is bigger), commissural vittae (2–)4–6, two of them, which are located near the carpophore are bigger than others. Commissural vittae are bigger than vallecular vittae. Endocarp formed of single line and narrow thin-walled long cells. Cell walls are lignified.

### 2.3. Etymology

The new species is named after the ancient name of the region where it grows.

### 2.4. Holotype

C4 Antalya Near Beyreli village 26 June 2015, 36°50′24.8′′ N, 32°22′14.41′′ E, M. Miski, E. Akalın & S. Anıl (ISTE 117051).

### 2.5. Localities

**Figure 4:**
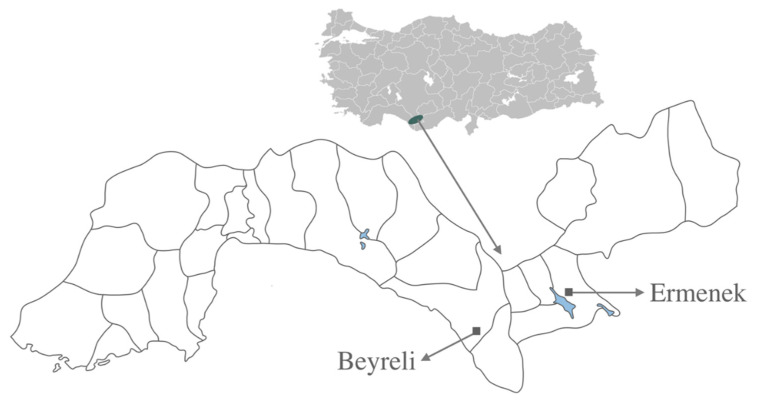
Distribution of *Ferula pisidica* in Turkey.

C4 Antalya Near Beyreli village 26 June 2015, 36°50′24.8′′ N, 32°22′14.41′′ E, M. Miski, E. Akalın & S. Anıl (ISTE 117051); C4 Karaman, Ermenek near Damlaçal, 25 June 2015, 36°40′43.6′′ N, 32°56′35.55′′ E, M. Miski & S. Anıl (ISTE 117074) (Figure 4). All specimens collected from the same locality and date are deposited and preserved under the same ISTE number. Specimens will be available upon request.

### 2.6. Ecology

The species is spread in stony, rocky areas and in openings of sparsely woodland in the valley. High plants are mainly, *Abies cilicica* subsp. *isaurica* Coode & Cullen and *Pinus nigra* subsp. *pallasiana* (Lamb.) Holmboe trees and also *Juniperus oxycedrus* L. scrubs; Small plants, *Dryopteris filix-mas* (L.) Schott, *Verbascum oreophilum* C. Koch, *Salvia candidissima* subsp. *occidentalis* Hedge, *Caucalis platycarpos* L., *Achillea setacea* Waldst. & Kit., *Astragalus pycnocephalus* Fisch., *Carduus nutans* L., *Vicia cracca* L., *Euphorbia macroclada* Boiss., *Galium aparine* L., *Nigella orientalis* L. are widespread in the area. The endemic species of *Erodium cedrorum* subsp. *salmoneum* (P.H. Davis & Roberts) P.H. Davis are observed.

### 2.7. Distribution and Proposed Conservation Status

*Ferula pisidica* is an endemic species to Southwest Anatolia (between A7 and A9 squares) and only known from two localities; therefore, it is considered as ‘Endangered’ (criterion B1 a).

It could also be categorized as ‘Endangered’ (criterion B2) for its known ‘area of occupancy’ of less than 500 km^2^, population size estimated to be fewer than 250 mature individuals (criterion C).

It is recommended that the species of *F. pisidica* should be placed under World Conservation Union (IUCN) threat category ‘Endangered’ (EN) [32].

## 3. Discussion

### 3.1. Chemotaxonomic Characteristics

Preliminary phytochemical investigations on the roots of *F. pisidica* indicate that majority of the secondary metabolites of this species are sesquiterpene esters that is a characteristic feature of *Ferula* species of subgenus *Peucedanoides*. Absence of the sesquiterpene coumarins and/or sulfur-containing compounds clearly excludes this species from subgenera *Scrodosma*, *Merwia*, *Narthex*, *Euferula* and *Dorematoides* Korov. Based on their similar morphological characteristics *F. haussknechtii* was identified as the closely allied species of *F. pisidica.* However, while the roots of *F. haussknechtii* exclusively produce apiene ester derivatives, sesquiterpenes with eleven membered monocyclic skeleton (Figure 1, compounds 7 & 10) [30], the roots of *F. pisidica* yield *cis-* and *trans-*daucane esters, sesquiterpenes with 5 and 7 membered bicyclic skeleton (Figure 1, compounds 1, 2, 4, 6 & 9). Since the biogenetic pathways of apiene and daucane esters were different [30,33] and chemotaxonomically differs from *F. haussknechtii* which confirms the new species status of *F. pisidica*.

### 3.2. Relationship

There were two sets of criteria employed to differentiate the new species from those of closely allied species; morphological characters (internal-external) and chemotaxonomical profiles. Based on these criteria, differences of the new species from those of closely allied species were tabulated in the Table 1. Amongst all potentially related species, *F. haussknechtii* and *F. brevipedicellata* were identified as the most closely related species. While *F. haussknechtii* could be differentiated by its shorter and slender habit, narrower and smaller sheaths, *F. brevipedicellata* has longer terminal leaf lobes ((3–)7–12 mm) and larger, and wider sheath. However, most distinct difference between the new species and those closely allied species was the length of basal leaves petiole; the length of new species basal leaves petiole was 20–32 cm long, in contrast *F. haussknechtii* basal leaves sessile and *F. brevipedicellata* basal leaves petiole length varies between 10–20 cm long. In addition, mericarps of the new species are smaller than related species except *F. elaeochytris.* No specific differentiation was observed between the leaf hair morphology of new species and aforementioned *Ferula* species. *Ferula* species examined are as follows:

*Ferula haussknechtii*: B9 Bitlis, Betweeen Tatvan and Gevaş districts, near Obuz (Kamer) village, Saya Ö. 7 October 1983, 2000 m. (ISTE: 109377).; B9 Van, Tımar district, 3 km. on the hill near the crossroads Saya Ö. 20 July 1984., 1840 m. (ISTE: 109388).; B9 Van, Erek Mountain, Rocky slopes with *Cachyris.* Davis, Peter Hadland & Polunin, Oleg Vladimir. 18 July 1954, 2800 m. (Edinburgh: E00262871).

*Ferula brevipedicellata*: C7 Malatya, Sürgü district, Eski Kurucaova village, near the arable field. Yildiz B. 13 May 1989, 1500 m. (ISTE: 105387).; B9 Bitlis, Hizan district, 19 km after Hizan to Pervari district. 2 km after the bridge. Saya Ö. 20 June 1983. 1060 m. (ISTE: 109423).; B9 Bitlis Hizan-Bahçesaray road 22 km, Sağıroğlu M. 9 July 2002. 1000 m. (Edinburgh: E00656571)

*Ferula elaeochytris*: C6 Hatay, Bezge border police station. Baytop A., Baytop T., 17 May 1962. (ISTE: 7096).; C6 Hatay, Kel (Akra) Mountain, south-east side. Tuzlaci E., 27 May 1977, 1400 m. (ISTE: 37198).; Turkey, Siehe W. 15 July 1985 (Edinburgh: E00175321).; Prov. Maras, distr. Goksun: Binboga dag; on N. E. side of Isik dag, Davis, P.H.; Dodds, L.G.; Cetik, D. 16 July 1952. 1900 m. (KEW: K001097251).

*Ferula rigidula*: B5 Kayseri, Yahyalı district, Sazak way, Baytop A. Tuzlaci E., 19 June 1977, 1200 m. (ISTE: 37615).; B9 Iğdir, Tuzluca district, between Hadımlı and Sanabdal villages. Altundağ E. 2 October 2008, 1280 m. (ISTE: 85835).; A9 Kars: Kağızman to Akçay (Aras valley) 1100–1200 m. Dry gravelly hills. Perennial. Davis, Peter H. 19 July 1966. (Edinburgh: E00175311).

*Ferula halophila:* B4 Konya, Yavşan memlehası near Tuzgölü, Saline Artemisia step. Davis&Dodds., 8 June 1952; (ISTE: 21109) B6 Kayseri, Sarız. district Between Sarız and Pınarbaşı 4 km. near the crossroads. Saya Ö. 22 June 1984. 1650 m. (ISTE:109382).; Prov. Konya Distr. Cihanbeyli, Tuz gölü, nr. Yavsan Memlehesi, Davis. 9 September 1949. (Edinburgh: E00002485).

*Ferula hermonis:* Adana, Saimbeyli district, Bozoğlan mountain above Obruk yayla. Davis, Dodds & Çetik. 7 August 1952. 2000 m. (Edinburgh: E00175317, E00175316)

## 4. Materials and Methods

This study is based on field work, literature surveys and herbarium materials. The new species material was compared to the herbarium materials of *Ferula* in Herbarium of Istanbul University Faculty of Pharmacy (ISTE), Royal Botanic Gardens Kew (K), Royal Botanic Garden Edinburgh (E). Comparative measurements of all species were taken from 10 mature individuals using suitable herbarium specimens. In addition, morphological analyzes of the new species were carried out on 15 specimens from the population at Beyreli village location and 10 specimens from the population at Ermenek location as well as on the living plants during the field studies performed at the aforementioned populations of two locations (i.e., Beyreli village & Ermenek, see Figure 4). Following the publication of current paper, isotype specimens of *F. pisidica* will be submitted to the additional herbaria in Istanbul (NGBB), Turkey and Edinburgh (E), UK.

Anatomical research material was dried so they were preserved in 70% ethanol. In this study at least 30 mature fruits of *F. pisidica* were analyzed. All transverse sections were cut by hand from the middle of the mericarps using a razor blade. Samples were examined in Sartur reagent (a compound reagent of Sudan III, lactic acid, aniline, iodine, potassium iodide, water, and alcohol) [34]. Photographs were taken with iPhone X. Measurements of mericarps were made by program ImageJ^©^. The fruit morphology and anatomy were described by using the terms of Botanical Latin [35], and Kızılarslan and Akalın [36].

## 5. Conclusions

*Ferula pisidica* from the ancient Pisidia province of Anatolia was described as a new species. The genus *Ferula* has some unusual Turkish and Iranian species that their phylogeny is not adequate to provide a decisive taxonomical information. The number of chromosome was found to be 2n = 22 in all investigated Turkish *Ferula* species, in addition, palynologic data was not used as a distinctive character for Apiaceae family. Therefore, sesquiterpenoid metabolite data of *F. pisidica* were used to corroborate the traditional taxonomical analyses. In addition to our extensive secondary metabolite knowledge on the Turkish *Ferula* species [23,24,25,26,27,28,29,30,31,34], distinctive internal and external morphological features, as well as chemical differences between the closely allied species provide sufficient evidence for the recognition of *F. pisidica* as a new species.

## Figures and Tables

**Table 1:** Comparison of diagnostic features of *F. pisidica*, *F. haussknechtii*, *F. brevipedicellata*, *F. elaeochytris*, *F. rigidula*, *F. hermonis Boiss*., *F. halophila Peşmen*.

Features	*F. pisidica*	*F. haussknechtii*	*F. brevipedicellata*	*F. elaeochytris*	*F. rigidula*	*F. hermonis*	*F. halophila*
**Stem length**	up to 250 cm	50–150 cm	100–250 cm	100–200 cm, terete	30–130 cm	100–150 cm, terete	60–90 cm
**Leaves**	5–7 pinnate, triangular ovate in outline, 25–40 × 30–85 cm	5 pinnate, triangular ovate in outline, 30–40 × 20–30 cm	5–6 pinnate, triangular ovate in outline, 30–65 × 20–45 cm	5–6 pinnate, triangular ovate in outline, 25–50 × 20–45 cm	5–6 pinnate, triangular ovate in outline, 15–30 × 10–25 cm	5–6 pinnate, triangular ovate in outline, 30–45 × 25–35 cm	5–6 pinnate, triangular ovate in outline
**Petiole (basal leaves)**	20–32 cm	sessile	10–20 cm	17–40(-48)	1–12 cm	-	8–10 cm
**Ultimate segments**	1–4 × 0.5–1.5 mm	0.5–3(–7) × 0.5–1.5 mm	(3–)7–12 × 1–2.5 mm,	1.5–2.5 × 0.5 mm,	1–5(–8) x 0.2–0.8 mm,	1.5–3 × 0.4 mm,	5–15(–20) × 0.4–0.8 mm,
	scabrid-setulose	densely setulose-puberulent	scabrid to glabrous	glabrous	scabrid	glabrous	very sparsely aculeolate-scabrid
**Sheaths**	Ovate 4–9 × 1–3 cm	Ovate-lanceolate 6–8 × 2–5.5 cm	Broadly ovate 7–12 × 5–8 cm	Ovate-oblong (6–)10–15 cm	Cylindric-oblong 3–12 × 1.5–3 cm	-	Ovate 6.5 × 7.5 cm
**Rays**	8–22	6–12	6–12(–18)	10–18	(4–)6–12(–15)	10–15	12–15
**Petals**	Yellow	Yellow	Yellow	Yellow	Yellow	Whitish-Green	Yellow
**Fruiting pedicels**	2–8 mm	(5–)7–10(–12) mm	0.5–6 mm	9–10 mm	(5–)7–15 mm	4–6 mm	5–9 mm
**Mericarps**	8–14 × 4–9 mm, oblong	13–16 × 6 mm, elliptic-oblong	8–14 × 4–7 mm, elliptic	9–12 × 4–5 mm, elliptic-oblong	10–12 × 6–7 mm, oblong to obovate	10–13 × 6–7 mm, elliptic-oblong	9–11 × 6–7 mm, obovate
**Lateral wings**	0.5–1 mm wide	0.5–1 mm wide	0.5–1 mm wide	0.5–1 mm wide	1–2 mm wide	1–1.5 mm wide	2 mm wide
**Dorsal vittae**	1–3 per vallecula	1 per vallecula	1 per vallecula	1 per vallecula	1 per vallecula	1(–2)	1 per vallecula
**Commissural vittae**	(2–)4–6	4	2–4	43,984	43,984	4–6	(2–)4
